# Shut the Door

**Published:** 1874-09

**Authors:** 


### "SHUT THE DOOR."

Easier said than done. There is scarcely anything in daily domestic life about which people show so little common sense.

If you find a door shut and have occasion to open it, close it afterwards; this rule holds for all seasons of the year, especially winter time.

There are several ways of shutting a door; a boor uniformly shuts it with a sling and a slam, jarring the whole side of the room and making the noise of a young cannon. Suppose the next room is occupied by a weary, suffering invalid, and that invalid was just falling into a sweet sleep, which the physician knows was the only partition separating from the grave, and this has been many a time the case, what then? The sleep was broken and the patient dies! In unnumbered cases the weary traveler, as he was just falling into a delicious sleep, has been waked up by persons coming to bed later at the hotel, by the harsh slamming of his door. Many times persons aroused from the condition of just falling into a sleep cannot get composed for hours after, and it may be to them the loss of a whole day. When you shut a door, take the knob in your hand, turn it back, close the door so gently that you can scarcely distinguish the sound yourself; let the knob go from your hand, and go off with the consciousness that you have disturbed nobody, have not roused up a weary traveler, and have not broken into the sweet sleep of some suffering invalid almost in the grave. We feel conscious while writing these lines that a million travelers will bless us for the suggestions made about "Shutting the Door."

